# Behavioral flexibility of the trawling long-legged bat, *Macrophyllum macrophyllum* (Phyllostomidae)

**DOI:** 10.3389/fphys.2013.00342

**Published:** 2013-11-25

**Authors:** Moritz Weinbeer, Elisabeth K. V. Kalko, Kirsten Jung

**Affiliations:** ^1^Institute of Experimental Ecology, University of UlmUlm, Germany; ^2^Smithsonian Tropical Research InstituteBalboa, Ancón, Panamá

**Keywords:** sensory ecology, aerial hawking, gleaning, bat echolocation, clutter, echo overlap

## Abstract

We assessed the behavioral flexibility of the trawling long-legged bat, *Macrophyllum macrophyllum* (Phyllostomidae) in flight cage experiments by exposing it to prey suspended from nylon threads in the air and to food placed onto the water surface at varying distances to clutter-producing background (water plants). The bat revealed flexibility in foraging mode and caught prey in the air (aerial hawking) and from the water surface (trawling). *M. macrophyllum* was constrained in finding food very near to and within clutter. As echolocation was the prime sensory mode used by *M. macrophyllum* for detection and localization of food, the bat might have been unable to perceive sufficient information from prey near clutter as background echoes from the water plant increasingly overlapped with echoes from food. The importance of echolocation for foraging is reflected in a stereotypic call pattern of *M. macrophyllum* that resembles other aerial insectivorous and trawling bats with a pronounced terminal phase (buzz) prior to capture attempts. Our findings contrast studies of other phyllostomid bats that glean prey very near or from vegetation, often using additional sensory cues, such as prey-produced noise, to find food and that lack a terminal phase in echolocation behavior. In *M. macrophyllum*, acoustic characteristics of its foraging habitat have shaped its sonar system more than phylogeny.

## Introduction

Species with a flexible use of behavioral strategies while hunting are likely to have access to more resources and exploit habitats better than species which are restricted to a specific foraging mode and hence a specific type of prey (Neuweiler, 1989, 1990). Generally, flexibility in foraging behavior often requires specific sensory adaptations as the bats may face different perceptual challenges imposed by different foraging modes. In addition, characteristics of the foraging habitat, in particular the relative position of food to the background clutter, strongly affect how bats find food, and determine the role of echolocation while foraging (Neuweiler, 1990; Schnitzler et al., 2003b).

Trawling bats, which collect insects or small fish from the water surface, such as *Noctilio* sp. (Noctilionidae) and some *Myotis* sp. (Vespertilionidae), are also known to hawk aerial prey and thus exhibit high flexibility in foraging behavior. This allows them to also take advantage of the insect-rich space above water bodies (Jones and Rayner, 1988, 1991; Schnitzler et al., 1994; Britton et al., 1997; Kalko et al., 1998). While trawling, the smooth water surface reflects most of the call energy away from the low flying animals and thus, little or no clutter echoes interfere with prey perception (Boonman et al., 1998; Siemers et al., 2001b). This leads, in conjunction with rather high sound intensities (Surlykke and Kalko, 2008) and despite high calling frequencies, to increased prey detection distances (Siemers et al., 2005). Foraging over water thus poses a perceptual task that is similar to aerial hawking of insects in open space. In both cases, echolocation represents the prime cue for finding and locating food.

The situation of trawling bats hunting over smooth water surfaces however strongly contrasts with bats that collect stationary food (gleaning) in cluttered environments such as fruits or insects next to vegetation. Gleaning bats face the sensorial challenge that clutter echoes often overlap target echoes (clutter overlap zone, Denzinger and Schnitzler, 2013), and thus frequently use additional sensory cues, in particular vision, olfaction, or prey-generated acoustic cues for finding food (e.g., Fenton, 1990; Fuzessery et al., 1993; Arlettaz et al., 2001; Schnitzler and Kalko, 2001; Altringham and Fenton, 2004). Most New World leaf-nosed bats (Phyllostomidae) are classified as gleaners as they typically take food close to or from surfaces in narrow space habitats near or within vegetation (Schnitzler and Kalko, 2001). Echolocation in Phyllostomids is primarily used for orientation in space and supplemented with additional sensorial information for finding food. Probably as an adaptation to cluttered environments, Phyllostomid bats emit rather uniform, short, high-frequency multi-harmonic and steep frequency-modulated (FM) broadband echolocation calls, which are well suited for measuring distances in confined space and to assess surface structures (Kalko and Condon, 1998; Thies et al., 1998; Kalko, 2004; Geipel et al., 2013). Previously, Phyllostomid bats have been mostly regarded as “whispering” bats with low sound intensities, but recent studies point toward much higher sound intensities associated with high signal directionality (Brinkløv et al., 2009). During target approach, the echolocation behavior of foraging phyllostomid bats differs from aerial insectivores as they do not emit a characteristic terminal phase or buzz prior to prey capture (a series of very short calls emitted at a high repetition rate; Neuweiler, 1989; Schnitzler et al., 2003b). Terminal phases of aerial hawking bats are thought to increase the information flow of moving prey, while reducing the overlap between emitted signals and returning echoes (signal overlap zone, Denzinger and Schnitzler, 2013), and minimize doppler-dependent ranging errors for prey localization (Holderied et al., 2008).

Unique among phyllostomid bats, the long-legged bat, *Macrophyllum macrophyllum* hunts over water (Harrison, 1975). The acoustic characteristics of this habitat resemble more (semi)-open than cluttered space as most signal energy is reflected away from the smooth water surface. In contrast to all other phyllostomid bats studied so far, the call pattern of trawling *M. macrophyllum* resembles that of aerial insectivorous and other trawling bats of different families (Jones and Rayner, 1988, 1991; Schnitzler et al., 1994; Kalko et al., 1998; Schnitzler and Kalko, 2001; Siemers et al., 2001a; Weinbeer and Kalko, 2007). While trawling for prey above open water it comprises a distinct search, approach and terminal phase (Weinbeer and Kalko, 2007).

In nature *M. macrophyllum* exhibits high flexibility in its foraging behavior. It mostly trawls insects from smooth water surfaces (Weinbeer et al., 2006), but was also observed catching insects in the air, as well as foraging close to banks of protruding water plants, *Hydrilla verticillata* (Hydrocharitaceae; Meyer et al., 2005). Presence of clutter-producing objects on the water surface however may affect prey perception by echolocation and reduce capture success due to effects of echo overlap (Schnitzler and Kalko, 2001) and the lack of an echo-acoustic ground effect (Zsebok et al., 2013). This has been shown previously in the insectivorous trawling bat *Myotis daubentonii*. To avoid overlap effects between echoes of prey and clutter, *M. daubentonii* changes its foraging strategy from trawling to aerial hunting, when the amount of clutter producing duckweed floating on the water surface reached a certain threshold (Boonman et al., 1998).

Here we investigate how the Neotropical leaf-nosed bat *M. macrophyllum* adjusts its flight and echolocation behavior according to sensorial challenges while trawling or aerial hunting. In particular, we assessed if background clutter elicit a behavioral change in foraging strategy—as known for aerial insectivorous bats—or a switch to other sensorial cues for prey detection—as it has previously been documented for most Phyllostomids.

If *M. macrophyllum* behaves like other trawling bats and continues to use exclusively echolocation for finding prey close to clutter, we hypothesize that capture success should decrease with proximity to vegetation. Furthermore, echolocation behavior should remain highly structured including search and approach calls and a terminal phase prior to prey capture. However, if *M. macrophyllum* behaves similarly to other phyllostomid bats, it should rather use other sensory cues such as prey-generated noise, vision or scent in a clutter situation. In this case, we expected *M. macrophyllum* to forage successfully even with prey close to or within clutter while omitting a distinct terminal phase.

To test these propositions, we presented prey under controlled experimental conditions in a flight cage to individual *M. macrophyllum* and assessed how proximity of food to horizontal clutter on the water surface affects foraging and echolocation behavior. Prey was offered to the bats either suspended in the air or placed onto the water surface at varying distances to clutter producing water plants. Finally, we compared echolocation and foraging behavior of the bat during the different tasks and between flight cage and field conditions to assess the influence of confined space onto call structure.

## Materials and methods

### Study animals

Over a period of 6 months (January–June 2003) we studied foraging and echolocation behavior of *M. macrophyllum* by conducting behavioral experiments in the flight cage and additional observations of free flying individuals on Barro Colorado Island (BCI), a field station of the Smithsonian Tropical Research Institute in Panamá. For behavioral experiments we caught nine adult (four females, five males) *M. macrophyllum* at a known roost site (Meyer et al., 2005). They were subsequently transferred into a flight cage (4.5 m × 4.5 m × 2 m) located inside the forest of BCI and kept individually for four consecutive nights each. Temperature, humidity, and noise level in the flight cage were similar to ambient values. After the behavioral experiments, all individuals were released back into the colony. In addition to these experiments, we also studied flight and prey capture behavior of *M. macrophyllum* in the field, foraging for ordinary prey under unaffected, natural conditions close (within 50 m) to their colony in a small cove next to the field station (for details see Meyer et al., 2005).

### Experimental setup in the flight cage

During behavioral experiments in the flight cage individual bats were exposed to prey (mealworms: larvae of *Tenebrio molitor*, Tenebrionidae) suspended in the air and on the water surface of a basin (3 m × 2 m) at varying distances to clutter-producing water plants. We chose mealworms as they come closest to one of the main foods of *M. macrophyllum* feeding mainly on small insects including water striders (pers. observations).

In the first set of experiments we tested the ability of *M. macrophyllum* to detect, classify and localize aerial prey using echolocation. We therefore suspended frozen (no movement, no scent) and live mealworms (wiggling, scent) on a thin (0.1 mm) nylon thread 20 cm above the water surface and recorded the bats' capture success. Experiments with mealworms suspended in air were arbitrarily interspersed by experiments with mealworms floating on the water surface (Weinbeer and Kalko, 2007) to impede accustoming of the bats to a particular situation.

**Figure 1:**
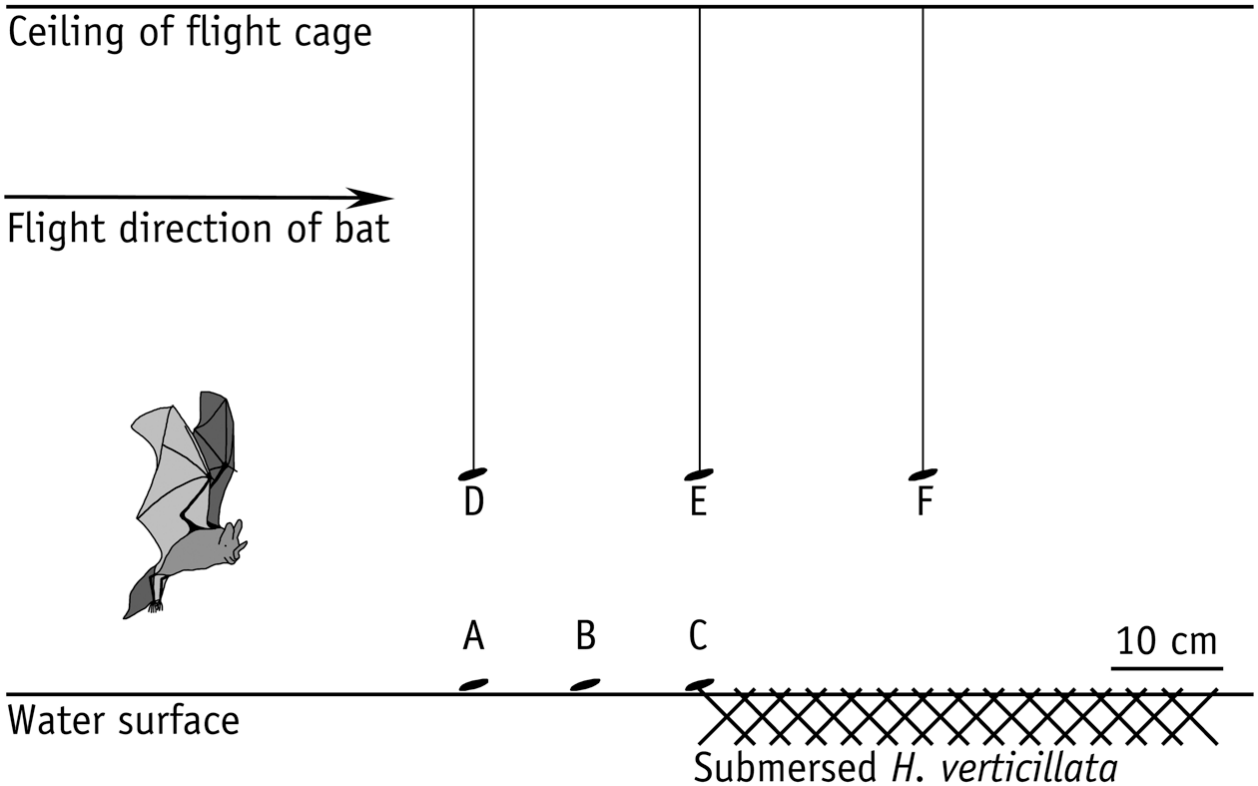
**Array of clutter experiments in the flight cage on BCI, Panamá, with *M. macrophyllum* searching for mealworms offered on the water surface (a–c) or tethered on a nylon thread (d–f) at different distances to horizontal clutter produced by leaves of *H. verticillata* water plants floating on the water surface**.

To assess the influence of clutter overlap on prey detection ability of *M. macrophyllum*, we conducted a second set of experiments, in which we exposed foraging bats to various amounts of clutter. We positioned a mat of about 0.5 m × 1 m of *Hydrilla verticillata* (Hydrocharitaceae) on the surface of the water basin. *Hydrilla* is a common water plant that regularly occurs within the foraging habitat of *M. macrophyllum* in Panamá. We conducted six different trials, in which we either placed mealworms onto the water surface at 20 cm, 10 cm, and 0 cm distance to the clutter-producing plants, or presented mealworms 20 cm above the water surface either at 20 cm in front of the *H. verticillata* bank, at the edge, or 20 cm over the clutter mat (Figure 1).

The full set of experiments was conducted first in randomized order for each bat and was then repeated several times, also in randomized order. Capture attempts were defined as successful when the bats directed their flight toward the mealworm, touched it, and subsequently removed it from the water surface or from the thread. Behavioral sequences were defined as unsuccessful when bats searched for food emitting search calls only, but passed the mealworm three or more times without any obvious behavioral attempt to approach and remove it. All behavioral experiments were conducted under low intensity of infrared light conditions, which is beyond the spectral range of vision in Phyllostomids (Winter et al., 2003).

### Analysis of flight and echolocation behavior during behavioral experiments and in the field

Flight behavior of bats during all experiments in the flight cage and all observations in the field was recorded with two CCD video cameras (Sanyo, VC 1950; resolution of half-frames: 20 ms) under infrared flash illumination. Simultaneously, echolocation calls of foraging *M. macrophyllum* were picked up by an ultrasound microphone, amplified and digitized (sampling rate: 312.5 kHz, 16 bit) with a custom-made system (Department of Animal Physiology, University of Tübingen, Germany). Calls were recorded at 1/15 of original speed onto a Sony Walkman professional (WM-DC6; Maxell XL-II 90 audiotape). Video sequences were synchronized with echolocation recordings (for details see Weinbeer and Kalko, 2007). For our analysis we randomly chose one video sequence per individual with a good signal-to-noise-ratio in the parallel acoustic recordings to avoid pseudoreplication. In total we thus analyzed nine video sequences, one of each bat hawking aerial prey in the flight cage, and eight sequences of bats in the field (originating from different individuals) with the program Simi Motion (Version 6.0, 2002, 85705 Unterschleißheim, Germany) for three-dimensional reconstruction of flight paths, speed, and bat-prey distance.

Analysis of echolocation call sequences was conducted using Avisoft SAS-Lab Pro (Version 4.2). Slowed-down signals were re-digitized (sampling rate 22.05 kHz), processed through a FFT, and displayed as color sonograms; spectrograms (FFT 512 points, Hamming window) were generated resulting in a frequency resolution of 646 Hz and a time resolution of 0.893 ms. Measurements were taken with a cursor on screen. We measured seven call parameters and limited our measurement to the second harmonic as it consistently contained the main signal energy of the multi-harmonic calls of *M. macrophyllum*. Based on sono- and oscillograms, we measured pulse duration [ms], pulse interval [ms] (difference between starting time of two consecutive calls), bandwidth [kHz], and peak frequency [kHz] (frequency at maximum amplitude). We also calculated repetition rate [calls/s] (number of calls per time unit), sweep rate [kHz/ms] (bandwidth divided by pulse duration), and duty cycle [%] (percentage of time in which signals are emitted). Measurements were taken at the point where call energy clearly exceeded background noise. This was at a minimum of 25 dB for search and early approach calls, sometimes declining to less (down to about 10 dB) for faint calls prior to capture.

For all statistical tests we used individuals as a statistical unit to avoid pseudoreplication. Herby, we only considered sequences with a good signal-to-noise-ratio and then randomly selected sequences for further analyses. For the first set of experiments, we chose two out of 14–21 echolocation sequences from each individual per experiment. We analyzed and compared flight and echolocation behavior during aerial hawking and assessed potential behavioral variability between hawking of live and dead mealworms. During our second set of experiments in order to assess the influence of clutter on echolocation behavior, we chose one echolocation sequence (out of 9–12) for each individual per trial. Finally, we selected 11 echolocation sequences (out of 65), recorded in 3 nights from bats foraging under natural conditions in the field. This reduced the possibility to include recordings of the same individual several times in the analysis. We then assessed differences in flight and echolocation performance between the confined space of the flight cage and the field. For more details see Weinbeer and Kalko (2007) and Brinkløv et al. (2010).

Following Schnitzler et al. (2003a), we described changes in echolocation behavior and correlated them with characteristic stages in foraging behavior. We thus discriminated between search calls (in *M. macrophyllum* usually regular groups of two calls, rarely a single call), approach calls (usually starting with a group of three up to seven calls and several subsequent groups of varying numbers of calls), and a distinct terminal phase or buzz emitted at a high repetition rate prior to capture (Weinbeer and Kalko, 2007).

To assess echolocation call parameters during foraging stages we calculated means per sequence over search and approach call parameters, respectively. For terminal phase calls however, which changed considerably over the course of the buzz, we separately analyzed the first call, the numerically median call, the call with shortest pulse interval (usually third to fifth last call), and the last call within the buzz sequence. In the first set of experiments with prey suspended in air, we used mean parameter values per individual of the two chosen sequences for statistical analysis to avoid pseudoreplication (Hurlbert, 1984). We then compared these results in a two-factorial Anova design (experiment * individual) with those of Weinbeer and Kalko (2007) to evaluate whether echolocation behavior differs between aerial hawking of tethered prey and trawling from the water surface, while accounting for individual differences in foraging behavior. For the second set of experiments we compared echolocation call parameters between experiments with a two-factorial Anova design (experiment * individual) to assess the influence of clutter on echolocation behavior of individuals. Finally, we compared our results in the flight cage with recordings from the free flying bats in the field in a two-factorial Anova design (experiment * individual) to assess potential differences in flight and echolocation behavior. All values are presented as mean ± *SD*.

## Results

### Foraging behavior

**Figure 2:**
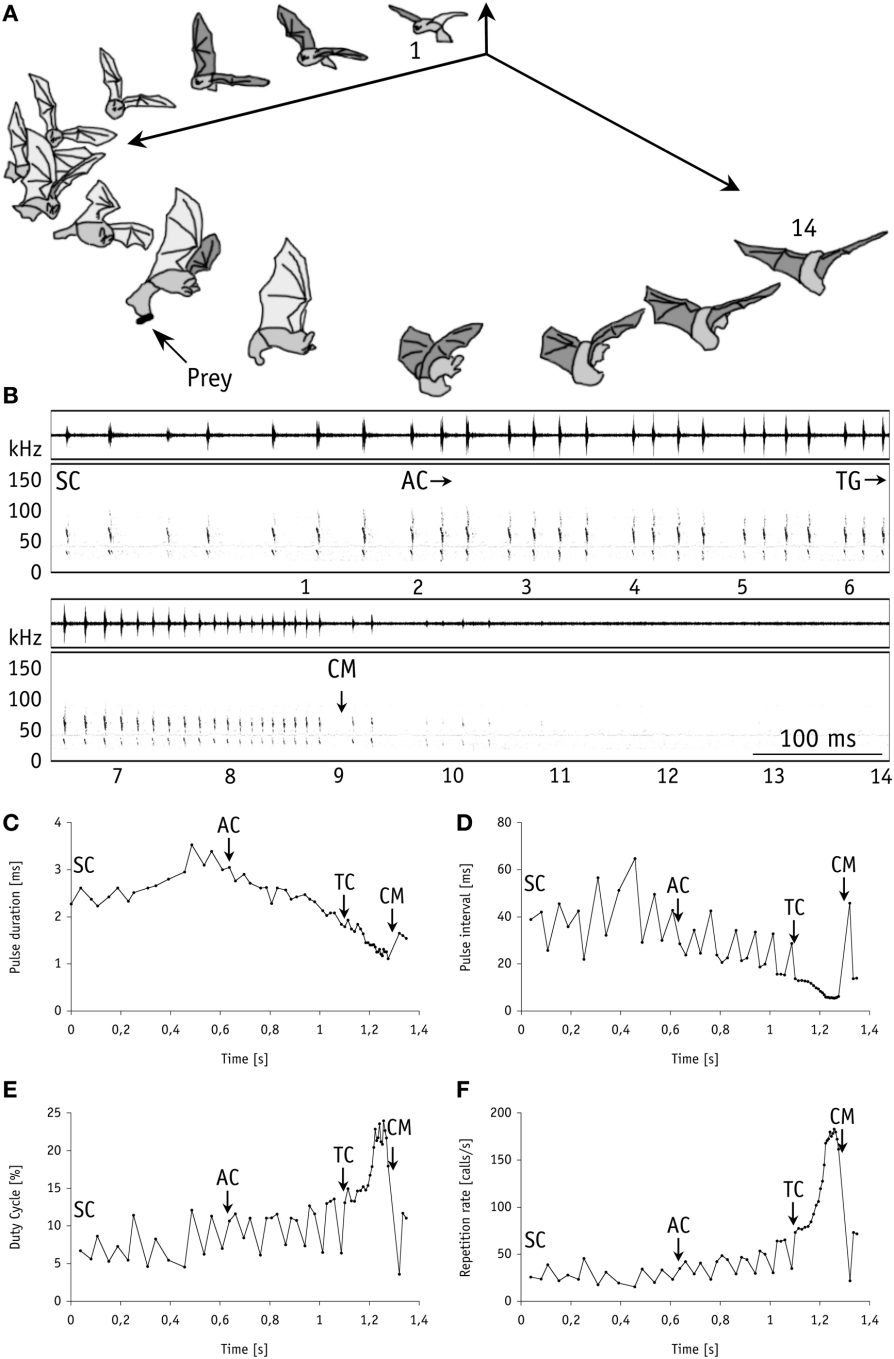
**Foraging behavior in 3-dimensional space synchronized with the corresponding echolocation sequence of *M. macrophyllum* approaching and capturing a mealworm suspended in the air above the water surface in the flight cage on BCI, Panamá. (A)** 14 images of the bat (temporal resolution: 80 ms). **(B)** Sonogram of the echolocation calls with time signal above; numbers below correspond to images of the bat. Plots of call parameters of the same echolocation sequence including **(C)** pulse duration, **(D)** repetition rate, **(E)** pulse interval, and **(F)** duty cycle. Abbreviations: SC, search calls; AC, start of approach calls; TG/TC, start of terminal group calls; CM, capture of mealworm.

In our first set of behavioral experiments all nine individual bats readily caught mealworms suspended in the air 20 cm above the smooth water surface (mimicking aerial prey). For unknown reasons, one bat took only a single tethered mealworm at the beginning of the experiments. We thus excluded it from subsequent analyses. In our experiments with live (*N* = 68) and with dead (*N* = 65) mealworms the bats removed them from the thread with 100% capture success. All individuals displayed a stereotypic echolocation behavior similar to trawling *M. macrophyllum* (Weinbeer and Kalko, 2007). When closing in on aerial prey, stages in foraging behavior were tightly linked with characteristic changes in echolocation behavior, with a pronounced shift from search to approach and a distinct terminal phase prior to capture (Figure 2). These results strongly suggest that, as it has been shown for trawling *M. macrophyllum* (Weinbeer and Kalko, 2007), echolocation is also the primary sensory cue used by this species to detect, classify, and localize aerial insect prey.

Assessing the influence live or dead aerial mealworms may have on echolocation behavior, we found no significant differences in echolocation call parameters (two-factorial Anova; all *F*_(1, 7)_ < 3.4, 0.1 < *p* < 0.96) between the two experiments, except for peak frequency of search calls, which was slightly higher in experiments with dead mealworms (55.9 kHz) than with live prey (55.1 kHz; *F*_(1, 7)_ = 7.5, *p* = 0.03). However, as this slight difference in frequency was within the range of the frequency resolution (645 Hz) of our analysis, we pooled all data of the two trials for further calculations (Table 1).

**Table 1:** **Seven echolocation parameters of *M. macrophyllum* foraging in the flight cage and in the field at BCI, Panamá**.

**Parameter**	**Habitat**	**Search**	**Approach**	**Terminal phase**			
				***F***	***M***	***S***	***L***
Pulse duration [ms]	Water	2.6 ± 0.3	2.3 ± 0.2	1.9 ± 0.2	1.5 ± 0.1	1.1 ± 0.1	1.0 ± 0.2
		1.9 − 3.6	1.9 − 2.7	1.5 − 2.4	1.2 − 2.0	0.9 − 1.6	0.7 − 1.5
	Air	2.5 ± 0.2	2.3 ± 0.1	2.0 ± 0.1	1.5 ± 0.2	1.2 ± 0.2	1.1 ± 0.2
		2.1 − 2.8	2.1 − 2.5	1.7 − 2.2	1.3 − 1.7	1.0 − 1.6	0.9 − 1.6
	Field	3.2 ± 0.7^**^	2.6 ± 0.2^**^	2.1 ± 0.3^*^	1.4 ± 0.3	1.0 ± 0.2^*^	0.9 ± 0.2^*^
		2.2 − 4.7	2.4 − 2.8	1.6 − 2.5	0.8 − 1.9	0.6 − 1.4	0.7 − 1.4
Pulse interval [ms]	Water	43.0 ± 11.5	19.9 ± 2.0	12.5 ± 1.7	8.1 ± 1.2	6.0 ± 0.5	
		22 − 74	16 − 23	9.0 − 16	6.1 − 11	5.3 − 7.6	
	Air	44.9 ± 14.9	19.1 ± 1.4	12.6 ± 1.2	8.0 ± 1.5	6.1 ± 0.6	
		29 − 81	17 − 22	10 − 14	6.3 − 11	5.4 − 7.6	
	Field	54.5 ± 11.9	17.9 ± 3.3	10.7 ± 1.8^*^	7.5 ± 1.8	5.8 ± 0.8	
		30 − 66	15 − 22	9.3 − 14	5.7 − 12	5.2 − 7.8	
Repetition rate [calls/s]	Water	24.8 ± 6.4	50.7 ± 5.3	81.5 ± 11.7	125.7 ± 17.6	166.7 ± 13.9	
		14 − 46	43 − 64	61 − 112	91 − 163	132 − 190	
	Air	24.2 ± 6.7	52.5 ± 3.7	80.3 ± 8.3	128.4 ± 21.8	166.6 ± 15.8	
		12 − 35	46 − 58	70 − 97	90 − 158	131 − 185	
	Field	19.4 ± 5.5	57.8 ± 11.4	96.2 ± 17.2	139.5 ± 27.6	174.6 ± 19.6	
		15 − 33	45 − 68	70 − 108	83 − 177	128 − 193	
Band-width [kHz]	Water	23.8 ± 1.8	25.9 ± 2.0	24.9 ± 2.6	21.7 ± 3.1	17.0 ± 2.7	15.0 ± 2.7
		21 − 28	21 − 30	18 − 30	15 − 30	12 − 24	9.9 − 22
	Air	23.7 ± 2.0	26.5 ± 1.6	25.8 ± 2.9	22.6 ± 2.7	18.9 ± 3.5	16.3 ± 3.5
		22 − 28	24 − 30	21 − 31	19 − 28	14 − 25	11 − 22
	Field	25.0 ± 2.4	26.9 ± 2.5	25.1 ± 3.1	21.0 ± 4.2	17.5 ± 2.2	16.4 ± 2.2
		20 − 30	23 − 30	20 − 31	11 − 26	14 − 20	13 − 20
Peak frequency [kHz]	Water	55.2 ± 2.4	54.9 ± 2.5	54.6 ± 2.8	54.4 ± 2.5	54.7 ± 2.5	53.6 ± 2.6
		50 − 59	50 − 60	50 − 63	48 − 59	48 − 58	48 − 57
	Air	55.3 ± 2.2	54.8 ± 2.2	55.0 ± 2.6	53.9 ± 2.0	54.5 ± 1.7	54.3 ± 2.6
		51 − 58	51 − 58	49 − 59	50 − 56	51 − 58	48 − 57
	Field	54.4 ± 1.7	51.9 ± 3.1^*^	51.9 ± 3.7^*^	51.7 ± 2.6^*^	53.3 ± 3.1	50.8 ± 5.3^*^
		51 − 56	47 − 57	47 − 59	48 − 56	47 − 57	43 − 56
Sweep rate [kHz/ms]	Water	9.4 ± 1.4	11.5 ± 0.8	12.9 ± 1.1	14.4 ± 1.2	14.9 ± 1.9	15.1 ± 2.2
		7.0 − 15	9.5 − 13	10 − 15	11 − 17	12 − 19	11 − 24
	Air	9.6 ± 1.3	11.6 ± 0.8	13.0 ± 1.1	15.2 ± 1.1	15.4 ± 1.6	14.5 ± 1.5
		8.0 − 13	11 − 13	11 − 15	13 − 17	13 − 19	12 − 17
	Field	8.0 ± 1.3	10.5 ± 0.9	11.9 ± 1.1	14.8 ± 1.6	17.3 ± 3.2	18.6 ± 4.1
		5.9 − 11	9.2 − 12	10 − 14	12 − 18	12 − 22	12 − 24
Duty cycle [%]	Water	7.0 ± 1.2	11.9 ± 1.2	15.8 ± 2.2	18.8 ± 2.5	19.1 ± 3.0	
		4.8 − 11	9.8 − 15	13 − 24	13 − 27	15 − 28	
	Air	6.9 ± 1.2	12.3 ± 0.8	15.9 ± 1.5	19.1 ± 3.4	20.5 ± 3.8	
		5.2 − 9.7	11 − 13	13 − 19	15 − 27	16 − 28	
	Field	7.0 ± 1.8	15.3 ± 2.3	20.1 ± 2.8	19.5 ± 4.3	18.0 ± 3.2	
		5.3 − 11	12 − 18	16 − 23	12 − 27	12 − 23	

Presented data (mean ± SD; min–max) are based on measurements taken from 715 search calls, 1610 approach calls, and 360 terminal phase calls of nine bats trawling mealworms floating on the water surface in the flight cage (Weinbeer and Kalko, 2007); 278 search calls, 579 approach calls, and 128 terminal phase calls of eight bats hawking tethered mealworms in the flight cage; and 100 search calls, 137 approach calls, and 44 terminal phase calls of 11 sequences of bats trawling floating natural prey in the field. Significant differences between captive bats and bats in the field are given as

* p < 0.05 and

** p < 0.001.

Abbreviations for terminal phase: F, first call; M, numerically median call; S, call with shortest pulse interval; and L, last call.

### Flight and echolocation behavior during target approach

Flight and echolocation behavior prior to detection of mealworms (search phase) was similar, whether prey was suspended in air or placed onto the water surface (Table 1, Figure 2). In fact, we found no differences in echolocation call parameters between aerial or trawling prey captures (two-factorial Anova; Tukey post-hoc comparison: all *p* > 0.06; *df* = 69) in our flight cage experiments. For detailed description of trawling behavior, see Weinbeer and Kalko (2007).

As indicator for prey detection we took the last search call prior to the beginning of approach calls (Weinbeer and Kalko, 2007). Aerial hawking *M. macrophyllum* detected mealworms at distances of 1–2 m (1.5 ± 0.3 m, *N* = 8). Similar to the observations of trawling *M. macrophyllum*, aerial hunting individuals emitted groups of three to seven approach calls with an inter-group interval of 36.1 ± 2.7 ms (*N* = 16). During their target-oriented approach flight at a speed of 2.4 ± 0.3 ms^−1^ (*N* = 8), bats clearly directed head, ears, and nose leaf toward the prey. In comparison to search calls, approach calls were characterized by decreasing pulse intervals, slightly shorter and decreasing pulse duration, increasingly higher repetition rate and duty cycle, and somewhat increased bandwidth and sweep rate (Table 1, Figure 2).

At a distance of half a meter or less (mean: 0.5 ± 0.1 m; 0.4–0.5 m; *N* = 8) toward the mealworms suspended in the air, *M. macrophyllum* started to emit a terminal phase of 23 ± 3 calls (range: 16–33 calls, *N* = 16 sequences) that lasted for 203.6 ± 33.3 ms (range: 127–307 ms; *N* = 16). Flight speed was slightly reduced to 2.2 ± 0.3 ms^−1^ (*N* = 8). The terminal phase calls were emitted at a very high repetition rate and characterized by short pulse duration, decreasing bandwidth, increasing duty cycle, and steep sweep rates (Table 1). After the first up to 10 buzz calls, *M. macrophyllum* entered the echo-overlap zone at a distance of 0.3–0.4 m (*N* = 8) from the prey, where calls began to overlap with echoes returning from prey (Figures 2, 4).

Just before prey capture, *M. macrophyllum* lowered its tail membrane, approximately perpendicular to its flight direction. It formed a pouch with its large tail membrane stabilized by its large feet, tail, and strong calcars. Head, nose leaf, and ears were directed throughout the approach toward the mealworm. Echolocation stopped a few cm in front of the food. As soon as the distal part of the tail membrane touched the suspended mealworm, the pouch was subsequently closed with the help of feet and calcars. Similar to removal of food from the water surface (Weinbeer and Kalko, 2007), feet and claws were not directly involved in the actual capture of suspended mealworms. The bat then wrapped the mealworm into its tail membrane and briefly pressed it against its abdomen. At that time, head, ears, and nose leaf were moved back into the upright position and the bat resumed echolocation. After the bat had taken the food with its mouth by bending its head quickly into the pouch, *M. macrophyllum* flew to a perch and ate it.

### Effect of clutter on foraging and echolocation behavior

The second set of experiments revealed that live prey (wiggling mealworms) on the water surface was equally well detected and removed by *M. macrophyllum* when placed at 20 cm (removal rate: 100%, *N* = 28 trials) and 10 cm (97%, *N* = 29 trials) in front of the water plants. However, capture success considerably dropped (23%, *N* = 31 trials) when prey was offered right at the edge (distance 0 cm) of the clutter plot of *H. verticillata* (Figure 1). In contrast, when mealworms were suspended 20 cm above the water surface in the air, bats had no difficulties in detecting them in front of (20 cm: 100%, *N* = 25 trials), at the edge of (0 cm: 100%, *N* = 24 trials), or 20 cm over the *H. verticillata* plot (93%, *N* = 28 trials). These results indicate that horizontal clutter of background vegetation negatively affected prey perception and capture success while trawling, but not during aerial hawking of *M. macrophyllum*.

We then compared echolocation behavior of bats in the experiments where mealworms were placed on the water surface at two distances to clutter-producing background (10 and 20 cm, respectively) to test for possible differences in signal parameters. We omitted data from the experiments with mealworms offered at the edge of the clutter plot for further comparison, as the bats only emitted search calls indicating that they had not detected food there. Overall, most call parameters did not vary significantly between the experiments (two-factorial Anova: *F*_(1, 8)_ < 6.2; 0.04 < *p* < 0.95; Table 2). Only the bandwidth of terminal phase calls with shortest pulse intervals was slightly narrower (14.4 kHz versus 15.8 kHz) in experiments with prey closer to clutter (*F*_(1, 8)_ = 9.4; *p* = 0.02), and pulse interval of search calls was somewhat longer (44.1 ms vs. 39.0 ms) in experiments with prey at larger distance to clutter (*F*_(1, 8)_ = 10.1; *p* = 0.01). However, as these differences were very small and close to the resolution of our analysis, we pooled all data for Table 2.

**Table 2:** **Seven echolocation parameters of nine *M. macrophyllum* foraging in the flight cage on BCI, Panamá**.

**Parameter**	**Search**	**Approach**	**Terminal phase**			
			***F***	***M***	***S***	***L***
Pulse duration [ms]	2.4 ± 0.3	2.1 ± 0.1	1.7 ± 0.1	1.4 ± 0.2	1.0 ± 0.1	0.9 ± 0.1
	1.9 − 2.8	1.9 − 2.2	1.6 − 1.9	1.1 − 1.7	0.8 − 1.3	0.8 − 1.2
Pulse interval [ms]	41.6 ± 10.5	17.9 ± 1.5	11.0 ± 1.2	7.9 ± 1.6	5.9 ± 0.7	
	24 − 62	15 − 21	8.9 − 14	6.2 − 11	5.1 − 7.8	
Repetition rate [calls/s]	25.6 ± 6.8	56.3 ± 4.9	91.9 ± 9.6	130.7 ± 22.8	172.3 ± 18.0	
	16 − 42	48 − 66	74 − 113	87 − 161	129 − 195	
Bandwidth [kHz]	22.9 ± 3.1	25.4 ± 1.9	23.5 ± 2.8	19.6 ± 2.6	15.1 ± 2.2	13.6 ± 1.7
	17 − 29	23 − 29	18 − 28	16 − 24	11 − 20	11 − 16
Peak frequency [kHz]	56.4 ± 2.2	55.4 ± 2.1	55.9 ± 2.7	54.8 ± 3.2	54.2 ± 2.8	53.2 ± 3.6
	53 − 60	50 − 58	52 − 62	47 − 59	49 − 59	47 − 58
Sweep rate [kHz/ms]	9.7 ± 0.9	12.3 ± 1.1	13.5 ± 1.5	14.5 ± 1.7	15.3 ± 2.4	15.2 ± 2.0
	7.9 − 11	10 − 14	11 − 16	12 − 18	10 − 19	12 − 19
Duty cycle [%]	6.4 ± 1.3	12.2 ± 1.2	16.0 ± 1.9	17.6 ± 3.0	17.2 ± 2.7	
	4.2 − 8.9	9.8 − 14	13 − 20	13 − 24	13 − 23	

Presented data (mean ± SD; min–max) are based on measurements taken from 85 search calls, 234 approach calls, and 72 terminal phase calls of bats trawling for mealworms exposed on the water surface near the clutter plot in two experiments. Abbreviations for terminal phase: F, first call; M, numerically median call, S, call with shortest pulse interval; and L, last call.

Calculation of the echo overlap zone (considering sound speeds of 346 m/s at 25°C) revealed that echoes of prey and background clutter overlapped in all trials in which mealworms were presented 10 cm or closer to *Hydrilla* (Figure 4). Overlap between background clutter and prey echoes also occurred in trials, where mealworms were 20 cm away from the water plants, if pulse duration exceeded 1.15 ms. Hence, when bats emitted search and approach calls, echoes of mealworms and clutter overlapped, while at the end of terminal phases calls were short enough to avoid overlap effects.

### Foraging and echolocation behavior in the field

**Figure 3:**
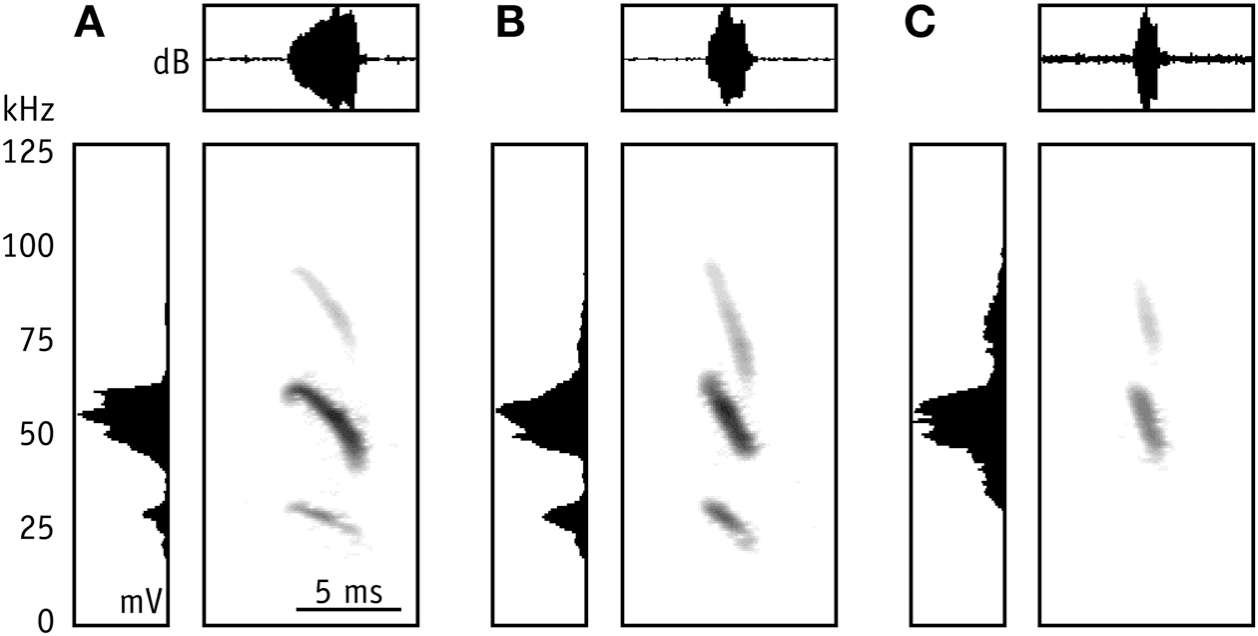
**Representative echolocation calls of *M. macrophyllum* foraging in the field above the water surface near the colony on BCI, Panamá.** Sonogram (time versus frequency) with oscillogram (time versus amplitude [dB]) above and averaged power spectrum (mV) to the left; values have been normalized. **(A)** Search call; note the short shallow-modulated onset of the call; **(B)** approach call; **(C)** terminal phase call, emitted prior to capture of prey.

**Figure 4:**
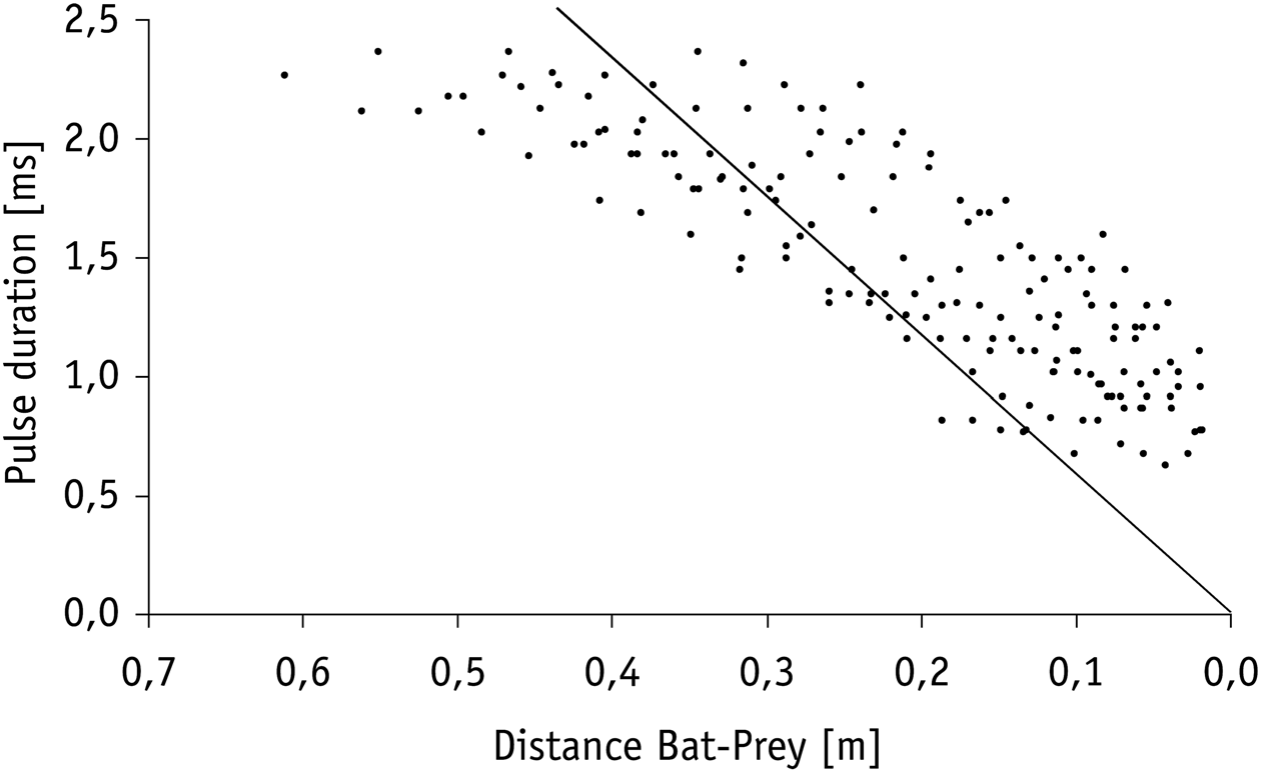
**Decrease in pulse duration of terminal phase calls from nine individuals of *M. macrophyllum* while approaching potential food floating on the water surface of Gatún Lake near the shore of BCI, Panamá.** The solid line indicates the limit beyond which calls overlap with returning echo from prey.

While searching for food under natural conditions in the field all bats flew at a higher speed (3.2 ± 0.3 ms^−1^, *N* = 6) than in the flight cage. Nevertheless, prey detection distance of 0.9–2.3 m (1.4 ± 0.5 m, *N* = 8) was comparable to the detection distance of floating or aerial mealworms measured in our flight cage experiments. After a brief pause of 46.3 ± 18.0 ms (*N* = 8 sequences), bats in the field began to emit groups of approach calls (Figures 2, 3) with an inter-pulse interval of 34.9 ± 4.6 ms (*N* = 7) similar to bats in the flight cage. Flight speed remained at 3.1 ± 0.5 ms^−1^ (*N* = 8). As in the flight cage, *M. macrophyllum* started to produce a distinct terminal phase (Figures 2, 3) composed of 19 ± 4 (range: 15–26, *N* = 11) calls and a mean duration of 153.6 ± 43.1 ms (99–245 ms, *N* = 11) at a distance of 32–61 cm (*N* = 8) after a short pulse interval of 27.7 ± 3.8 ms (*N* = 8). Flight speed remained high (3.0 ± 0.5 ms^−1^, *N* = 8). After up to 12 buzz calls, *M. macrophyllum* entered the echo overlap zone at a distance of 21–37 cm to the prey with a pulse duration of 1.8 ± 0.3 ms (*N* = 8), where echolocation calls started to overlap echoes returning from prey (Figure 4). A few cm before bats reached the food, echolocation stopped for a period of 41.4 ± 13.3 ms (*N* = 8), during which *M. macrophyllum* took the prey from the water surface. Subsequently, the bats resumed echolocation.

We found several significant differences in call parameters between bats recorded in the flight cage and in the field (two-factorial Anova; all *F*_(2, 69)_ < 15.5; Tukey post-hoc comparison: all *p* > 0.0001; Table 1). As a general pattern, pulse duration of search and approach calls was longer in the field than in the flight cage, while terminal phase calls were shorter. Additionally, in the field search and approach calls were emitted at longer pulse intervals, while pulse interval between terminal phase calls was shorter than in the flight cage. Finally, echolocation calls in the field were always emitted at lower peak frequencies than in the flight cage, while bandwidth did not vary significantly (Table 1) and most search calls of bats from the field started with a very short, shallow-modulated component (Figure 3).

## Discussion

### Foraging behavior of *M. macrophyllum*

According to our results, *M. macrophyllum* uses echolocation as the prime sensory cue for finding prey. Furthermore and in reference to our observations in the field, our behavioral experiments revealed that *M. macrophyllum* detects and captures prey equally well in trawling (Weinbeer and Kalko, 2007) and in aerial hawking mode. This behavioral flexibility parallels observations of a variety of trawling bats. The larger of two *Noctilio* species that occur in sympatry with *M. macrophyllum*, *N. leporinus*, trawls prey from the water surface (e.g., Schnitzler et al., 1994) and occasionally performs aerial captures (Übernickel et al. subm.), while the smaller *N. albiventris* frequently forages in trawling and aerial hawking mode (Kalko et al., 1998). Likewise, another sympatric species, the small proboscis bat, *Rhynchonycteris naso* (Emballonuridae), mainly feeds on aerial prey above water bodies, but also takes insects directly from the water surface (unpubl. data). Also various *Myotis* species are known to trawl and to hawk insects in the air (e.g., Britton et al., 1997; Jones and Rayner, 1988, 1991; Kalko and Schnitzler, 1989). The ability of trawling bats to switch their foraging strategy from trawling to aerial hunting while maintaining echolocation as the sole sensorial modality is very likely linked to the similarity of perceptual tasks. Perceptually, foraging above water is rather similar to aerial hawking in (semi-)open space, as the smooth water surface reflects most of the call energy away from a low-flying bat. Hence, over water and in (semi-)open space, little or no clutter echoes interfere with prey perception by echolocation (Boonman et al., 1998; Rydell et al., 1999; Siemers et al., 2001a).

Thus our results confirm that, as it has been shown before in trawling *M. macrophyllum* above open water areas (Weinbeer and Kalko, 2007), echolocation behavior of *M. macrophyllum* strongly resembles echolocation behavior of other trawling and aerial hawking bats (e.g., Jones and Rayner, 1988, 1991; Kalko and Schnitzler, 1989; Schnitzler et al., 1994; Kalko et al., 1998; Zsebok et al., 2013), even when foraging close to background clutter. It however markedly differs from echolocation behavior of other phyllostomid species that typically glean food from vegetation.

Moreover, the distances toward prey at which echolocation behavior of *M. macrophyllum* by changing from search to approach calls reflects target detection (reaction distance), as well as the onset and duration of the terminal phase are similar to those observed in the trawling vespertilionid bat, *Myotis daubentonii*, and aerial hawking pipistrelle bats, *Pipistrellus sp*. (Kalko and Schnitzler, 1993; Kalko, 1995). However, in contrast to other trawling and aerial hawking bats, *M. macrophyllum* enters the signal-echo-overlap zone already at about a distance of 40 cm to prey items. In *M. daubentonii* (Kalko and Schnitzler, 1989) and *P. pipistrellus* (Kalko and Schnitzler, 1993), echolocation stops before entering the echo-overlap zone, as the bats enter this zone only at distances of about 10–20 cm before reaching their prey. This suggests a higher overlap tolerance of phyllostomid echolocation calls with regard to clutter which might be due to a relatively high bandwidth of buzz calls, which potentially facilitates separate processing of call components. A high bandwidth reduces or might prevent potential signal-echo overlap as echoes of the broadband calls can probably be processed in the bat's hearing system as many frequency bands in different channels (Wiegrebe and Schmidt, 1996; Siemers and Schnitzler, 2004; Weinbeer and Kalko, 2007).

### Effect of clutter on echolocation and foraging behavior

Detection performance of trawling *M. macrophyllum* declined with decreasing distance between the mealworm and the clutter and hence increasing effects of the clutter overlap. As long as prey echoes were only slightly overlapped by clutter echoes (≥10 cm distance of prey to clutter), bats were able to find the mealworms, while prey that was completely buried within clutter-producing background could no longer be detected. This perceptual difficulty was well reflected in echolocation behavior, as *M. macrophyllum* did not emit approach calls and a terminal phase when prey was buried in clutter. Our findings are in accordance with results of *M. daubentonii* for which detection performance decreased with increasing clutter (Zsebok et al., 2013) and ceased foraging in low flight when the cover with duckweed on the water surface became too dense (Boonman et al., 1998).

Interestingly, *M. macrophyllum* did not use other sensory cues to perceive prey buried in clutter, as is typical for most phyllostomid bats. Perhaps, echolocation call structure of the broadband, steep FM calls like those emitted by *M. macrophyllum* increases overlap tolerance. Corresponding neuronal filters are likely to analyze those bands separately, each of which has a shorter duration than the complete call, thus reducing the echo overlap zone (Wiegrebe and Schmidt, 1996). Evidence for this proposition comes from a study of five species of *Myotis* where the tight link between bandwidth and vertical clutter tolerance had been studied (Siemers and Schnitzler, 2004). Species like *M. nattereri* with short, steep FM search calls and a very broad bandwidth of 120 kHz had no difficulty (capture rate: 100%) in finding prey presented at a distance of 5 cm to clutter. In contrast, *M. dasycneme* or *M. daubentonii*, which emit calls of lower bandwidth (44 kHz and 57 kHz, respectively), caught 100% of the offered prey only at 25 cm distance to clutter (Siemers and Schnitzler, 2004). As Zsebok et al. (2013) pointed out, it seems of no relevance to target detection and prey capture attempt whether the clutter producing surface is vertically or horizontally oriented. Our results show that *M. macrophyllum* fits well into this pattern, as it was able to find most prey (capture rate: 97%) at a distance of 10 cm relative to clutter, emitting search calls with a total bandwidth of approximately 70 kHz (Weinbeer and Kalko, 2007). However, while main call energy in *Myotis* was concentrated in the first harmonic, *M. macrophyllum* emitted calls of three and occasionally up to four harmonics (Weinbeer and Kalko, 2007). This may permit *M. macrophyllum* to integrate echo information over several harmonics.

### Evolution of flexibility in foraging behavior

Flexibility in foraging behavior while maintaining echolocation as the sole sensory mode is likely to grant *M. macrophyllum* access to a wider range of prey, including insects sitting on the water surface or flying somewhat above water. As an example, *M. macrophyllum* often feeds on an abundant, introduced moth, *Parapoynx diminutalis*, (Pyralidae). Its larvae develop in *H. verticillata* plants, where at certain times of the year numerous imagoes emerge. We frequently found scales of *P. diminutalis* in the feces of *M. macrophyllum* (Meyer et al., 2005; Weinbeer et al., 2006). Moths are taken directly from the water surface or caught in mid-air. This efficient exploitation of particular resources based on flexibility in foraging behavior has been found for a wide number of insectivorous bat species that regularly switch between aerial hawking and gleaning from (rough) surfaces (e.g., Schumm et al., 1991; Arlettaz, 1996; Chruszcz and Barclay, 2003).

Foraging flexibility in *M. macrophyllum* may finally be seen in an evolutionary context together with its associated prey detection mode and echolocation behavior. In a postulated evolutionary scenario, extant bats are descended from a late echolocating aerial hawking insectivorous bat. However, it remains unclear whether some groups, such as phyllostomid bats, may have switched several times between aerial hawking and gleaning mode close to or within vegetation (Schnitzler et al., 2003b; Simmons and Geisler, 1998). As most extant leaf-nosed bats produce echolocation calls that are primarily used for spatial orientation, and as they forage mostly in narrow space habitat that hampers use of echolocation for finding food close to or on surfaces, we postulate that *M. macrophyllum* has evolved from this group in a rather unique manner (e.g., Fuzessery et al., 1993; Schnitzler and Kalko, 1998, 2001; Rydell et al., 1999; Arlettaz et al., 2001; Jones et al., 2003).

Indeed, based on molecular data, *M. macrophyllum* is placed near highly derived phyllostomid genera (*Lonchorhina*, *Macrotus*, *Mimon*, or *Trachops*; Freeman, 2000; Wetterer et al., 2000; Lee et al., 2002) that all show the typical, rather uniform echolocation behavior of phyllostomid bats gleaning food within cluttered habitats without emitting a distinct terminal phase. Furthermore, similarities in echolocation and foraging behavior among largely unrelated trawling bats strongly suggest that both foraging and echolocation behavior have evolved independently several times in several families in response to similar ecological conditions rather than *M. macrophyllum* representing a “primitive” form of the Phyllostomidae.

From our experiments in the flight cage and observations in the field we infer that *M. macrophyllum* uses echolocation as a prime sensory mode for finding prey and argue, that this reflects an adaptation to the acoustic characteristics of its main foraging habitat (over water). In addition, *M. macrophyllum* revealed a high flexibility in foraging behavior (trawling and aerial hawking), which is astonishingly similar to other trawling bats. Beyond this, our acoustical analysis showed that *M. macrophyllum* is able to tolerate echo overlap to a certain degree, particularly, when prey is partially buried within clutter. These sensory adaptations attribute *M. macrophyllum* a unique position among leaf-nosed bats, and strongly suggest a convergent evolution of its echolocation behavior with that of other trawling and aerial hawking bats. Thus, in its sensory adaptations, *M. macrophyllum* rather resembles distantly related trawling and aerial hawking bats than closely related Phyllostomids.

## Conflict of interest statement

The authors declare that the research was conducted in the absence of any commercial or financial relationships that could be construed as a potential conflict of interest.
